# The fourth controlled release society workshop on oral peptide administration

**DOI:** 10.3389/fddev.2026.1860678

**Published:** 2026-06-01

**Authors:** David J. Brayden, Stephen T. Buckley, Andrew L. Lewis

**Affiliations:** 1 Advanced Drug Delivery, University College Dublin, Dublin, Ireland; 2 Oral Delivery Technologies, Drug Product Research, Therapeutics Discovery, Novo Nordisk, Denmark; 3 Quotient Sciences, Nottingham, United Kingdom

**Keywords:** ingestible devices, intestinal permeability, oral peptide delivery, peptide clinical trials, permeation enhancers

## Abstract

The Controlled Release Society (CRS, www.controlledrelease.org) held its fourth iteration of research and development in oral peptide administration on 14 July 2025 in Philadelphia (United States), entitled “Recent advances in oral peptide delivery: from molecule to market.” The previous versions were held in 2008, 2014, and 2023 (online), so it is a topic of continuing interest to drug delivery scientists as several oral peptide products have been released in the market over the period despite the enormous technical and commercial challenges involved. The workshop was co-sponsored by Frontiers in Drug Delivery, Novo Nordisk, Johnson and Johnson, Merck, Protagonist Therapeutics, Quotient Sciences, Genentech (Roche), and Bristol-Myers Squibb. It consisted of nine talks and two roundtable sessions. The overall aim of the workshop was to provide an update on the current clinical oral peptide programmes, address mechanistic progress on permeation enhancers, and evaluate the limitations of preclinical bioassays and animal models.

David Brayden (University College Dublin, Ireland) gave the opening talk and discussed the current trends in the field by recapping on the significance of the approvals of oral semaglutide (Rybelsus™; Wegovy® pill) and oral octreotide (Mycappsa™) and updating the clinical trial progress of the oral administration of the macrocyclic peptides, MK-0616 (enlicitide decanoate, New Drug Application submitted), JNJ-2113 (ICOTYDE™ (icotrokinra) approved in 2026), and LUNA18 (Phase I, terminated), amongst others (Table 1). He discussed the rationale for oral administration in the context of long-acting weekly subcutaneous (SC) injections for the same molecule, as is the case with the glucagon-like- peptide-1 analogues where patient preferences for the convenience of oral administration can be clouded by strict oral dosing requirements (Boye et al., 2021). Based on this example, a conservative view of the benefit of oral administration is that it offers an alternative route for some patients, while others may opt to continue with weekly injectables. Despite this caveat, oral administration of peptides is the favoured administration option for most patients.

**TABLE 1:** Clinical development of oral peptide formulations for systemic administration (2026).

Company	Peptide	Description	Formulation	Phase
Novo Nordisk	Semaglutide (Rybelsus®, Ozempic (R) oral, and Wegovy® oral)	Lipidated long half-life GLP-1 analogue. Injectable to oral	SNAC tablet	Three approvals since 2019
Chiasma	Octreotide	Injectable to oral	C_8_ in oily suspension	Approved (2020)
Janssen	JNJ-2113 macrocycle (Icotrokinra™)	Scaffolds and phage display for anti-IL-23 peptide	No PE	Approved (2026)
Merck	MK-0616 macrocycle (Enlicitide™)	mRNA display for anti-PCSK9 peptide	C_10_	III, NDA submitted (2026)
Enteris	Leuprolide	Peptelligence™ enhancers	Acyl carnitines/citric acid	II; ongoing
Oratech JV	Insulin (ORMD-0801)	POD™ enhancers and peptidase inhibitors	EDTA, SBTI, and bile salts	II; completed
Viking	VK2735 (GLP-1/GIP RA)	Permeation enhancer	C_10_ and SNAC in WO 2024/192,219	II (2025)
AstraZeneca	MED17219 (GLP-1 RA) lipidated peptide	Permeation enhancer	Chenodexoycholate/Propyl gallate	(I, 2023, terminated)
Chugai pharma	LUNA18 (RAS inhibitor)	mRNA display and medicinal chemistry	No PE	I (2025, terminated)
Novo Nordisk	Amycretin (GLP-1/amylin agonist)	Permeation enhancer	SNAC	I (2025)
Sciwind Biosciences	XW004, Ecnoglutide (acylated GLP-1 RA)	Permeation enhancer	Not disclosed	I (2024)
Rani	Octreotide, PTH	Robotic pill	Device	I; (2020)

Abbreviations: PE (permeation enhancer), SNAC (salcaprozate sodium), C_10_ (sodium caprate), C_8_, (sodium caprylate), PTH (parathyroid hormone), GLP-1 (glucagon-like peptide 1), GIP (glucose-dependent insulinotropic polypeptide), RAS (rat sarcoma oncogene), IL-23 (interleuken-23), SBTI (soybean trypsin inhibitor), EDTA (ethylenediaminetetraacetic acid), POD™ (protein oral delivery), PCSK9 (proprotein convertase subtilisin/kexin type 9). The FDA approved the oral small molecule GLP-1 receptor agonist analogue orforglipron (Foundayo™) in April 2026.

In addition, the lack of prediction of preclinical animal models for human oral dosage performance with intestinal permeation enhancers was also addressed. For example, aside from the difference in gastrointestinal physiology between the gut regions in distinct species, aqueous mixtures perform differently in rat models than solid dosage forms in dogs or macaques, while cell-based systems cannot tolerate the high concentrations of excipients that are typically presented *in vivo*. Brayden’s view was that the *in vivo* rat gut instillation models in particular are informative despite their limitations, and they can provide useful clues for the direction to follow in large animal models. He reviewed the current thinking about the mechanistic comparisons between the gold-standard medium fatty acid-based permeation enhancers, namely, salcaprozate sodium (SNAC) and sodium caprate (C10). These two enhancers are of immense importance to the field as SNAC has the status of generally regarded as safe (GRAS) excipient, while C10 has a history of use in phase II and phase III clinical trials. Brayden described how molecular dynamic simulations can model how both these agents interact with plasma membranes in the presence of mixed micelles and bile salts present in the GI tract (Hossain et al., 2020). With cryo-EM revealing micelles and vesicles of C10 at different pH values that are present in the simulated fluid of gut regions (Cunha et al., 2026), it is clear that our understanding of the enhancer–peptide relationship is presented at the epithelium, and the subsequent path that the peptide takes in traversing it is still rudimentary. Other aspects discussed were whether any other enhancers could compete with SNAC and C10. Here, in a project supported by Gattefosse Ltd (France), he highlighted published data on Labrasol®, Labrafac™, and Capryol-90™, where the medium-chain fatty acid glyceride components could enable blood levels of insulin in a rat model that were equivalent to those seen with C10 (McCartney et al., 2025). A part of the attractiveness of these solubilising excipients is their good safety profile in approved products and the capacity to be repurposed as enhancing excipients in capsule formulations of peptides. Other topics generating audience questions were whether enhancers can be combined and the optimal release kinetics of peptides and enhancers in tablet formulations in the stomach or small intestine.

Tahnee Dening (Genentech, United States) described macrocyclic peptide drug interactions with bile salts and biorelevant colloids using advanced nuclear magnetic resonance (NMR) and artificial membranes. Focussing on octreotide, a low permeability and high solubility peptide, she addressed how it interacts with bile salts present in the GI tract, as mimicked by the biorelevant buffers, fed-state simulated small intestinal fluid (FeSSIF), and fasted-state simulated small intestinal fluid (FaSSIF). According to Dening’s studies from her post-doctoral work, bile salts can strongly interact with aromatic and lysine amino acid residues in peptides to form colloids, micelles, and mixed micelles depending on their critical micellar concentrations (CMC) in particular buffers (Dening et al., 2021). Dihydroxy bile salts appear to impede the peptide flux across membranes to a greater extent than trihydroxy versions; yet, they offer good protection against peptidases. NMR studies highlighted the interaction between bile salts and amino acid residues on octreotide, and the resulting complexation occurred more readily for octreotide than for a comparator peptide lacking such residues, such as desmopressin (Dening et al., 2025). Bile salts are surfactants with low CMC values, but whether they can increase peptide absorption from the GI tract is complicated by their interaction potential, at least with some peptides. This is contrary to what might typically be predicted flux-wise for surfactant-type permeation enhancers.

Stephen Buckley (Novo Nordisk) discussed the barriers to oral macromolecule delivery and made some predictions on what peptide molecules might be developed next for this administration route. On patient preferences, he emphasised that oral administration is advantageous over other routes for chronic indications and because there may be pharmacological advantages in routing to the liver via the portal vein rather than flooding the peripheral circulation. In reviewing the development of Rybelsus® (oral semaglutide), a major outcome was the combination of improving the potency and reducing the clearance of the glucagon-like-peptide-1 (GLP-1) analogue and then matching it with an oral permeation-enhancer technology (SNAC), thereby yielding an oral product that achieved similar pharmacologic endpoints for patients with type II diabetes as the injectable counterpart despite its low bioavailability. He described the recent oral formulation efforts to additionally develop amycretin, a dual GLP-1 and amylin receptor peptide agonist that has led to weight loss and acceptable safety endpoints in clinical trials in patients with obesity following weekly SC-administered doses of up to 50 mg (Gasiorek et al., 2025). In looking ahead, he reviewed the bioinspired device attempts to achieve double-digit bioavailability in preclinical studies but emphasised the uncertain regulatory path for obtaining approval along with patient acceptability. Since the workshop, a higher-strength oral semaglutide of 25 mg (Wharton et al., 2025) was approved by the FDA for patients with obesity (Wegovy® Oral), along with a second-generation Rybelsus® (Nielsen et al., 2025) with lower semaglutide doses for patients with type II diabetes.

Ricardo Diaz de Leon Ortega (Quotient Sciences, United Kingdom) discussed the applications of physiologically based pharmacokinetic (PBPK) modelling in oral peptide delivery. He defined PBPK as an approach that integrates compound (physicochemical) and *in vitro* drug metabolism and pharmacokinetics (DMPK) and formulation properties (e.g., dissolution) with physiological parameters (e.g., tissue volumes and blood flow) through a set of equations. Simulations are then devised to see if they can describe the observed data and, if not, the model from which the simulation is refined until it can accurately reflect the dataset. The benefits include better understanding of processes, including the formulation and performance of permeation enhancers, outcome predictions, and a guide to better experimental design. Two unpublished cases were then presented, where the first was a retrospective study using information examining SNAC as an enhancer in 300 mg and 600 mg concentrations in enteric-coated tablets with a poorly permeable peptide supplied by Bristol Myers Squibb. A Weibull model was used to fit dissolution data, and PK-Sim® and MoBi® were used for simulations, with dog and human PK data supplying the input datasets. The key conclusions were that enhancers performing as functional excipients need to be included in the dissolution profiles and in plasma measurements to better inform the model. Dissolution profiles from enteric tablets should be conducted in biorelevant media and, even then, approximation to *in vivo* dissolution is sub-optimal, and this weakens the model. Regarding SNAC’s mechanism, interaction with small intestinal epithelia with this peptide indicated a complex process with channels or pores created through fluidisation in the plasma membrane, mimicking some aspects of paracellular flux. In the second example, modelling and simulation were used to explore the potential interactions of oral semaglutide (Rybelsus®) with 10 therapeutic molecules, including metformin and furosemide. The impetus for this work was because semaglutide has the potential to slow gastric transit time and the formulation contains the enhancer SNAC, so one or both of these features could affect the PK of a range of co-administered small molecules from across the biopharmaceutical classification system (BCS). The conclusion was that the potential interactions were dependent on the ‘victim’ molecule’s solubility, permeability, ionisation state, and whether there is any saturable metabolism and/or transport in the gut. Notable predictions from PKPD modelling were an increase in oral bioavailability from 33% to 43% for metformin and from 49% to 59% for furosemide in the presence of semaglutide, whereas other examples yielded no changes or a slight increase, with only one predicting a decrease. However, whether the interactions might be clinically important depend on the therapeutic index of the ‘victim’ molecule.

Thomas Von Erlach (Vivtex Corporation, United States) discussed the pre-clinical screening of permeation enhancers for oral peptide administration in oral dosage forms using GI-ORIS™ technology (Von Erlach et al., 2020). An important topic for the field is the lack of predictive power of current preclinical models pertaining to the performance of permeation enhancers in oral peptide formulations. Von Erlach described a workflow whereby porcine small intestinal tissue mucosae can be mounted in a robotic Transwell-like plate to create mini-Ussing chambers for screening multiple enhancers and combinations thereof with selected peptides. AI can be used to find the enhancer properties for particular peptides, while screening in GI-ORIS can be evaluated for liquid formulation in rat models before being formulated in a solid dose for testing on large animals. He discussed the translational challenges in moving from liquid to solid dosage forms for oral peptides. Nonetheless, a case study for oral vancomycin was presented where a solid dose formulation was designed following five iterations that delivered more peptide than a C10-based formulation in dogs. A proprietary immediate-release tablet formulation with an undisclosed enhancer composition also outperformed Rybelsus® in a dog PK study. However, success in a large animal model does not ensure success in humans, and this was exemplified by a published study from Novo Nordisk researchers where a proprotein convertase subtilisin/kexin type 9 (PCSK9) inhibitor peptide could successfully be delivered by a combination of C10, SNAC, and diluent from an immediate-release (IR) tablet in dogs but failed in humans (Niu et al., 2025). This highlighted gaps in knowledge of the optimal *in vivo* release profiles and accurate estimates of concentrations of peptide and enhancers in different gut regions between species, along with their interaction with GI luminal contents. Returning to the GI ORIS™ system, Von Erlach showed data confirming that the orally bioavailable macrocycle RAS inhibitor, LUNA18, indeed had high basal permeability in the *ex vivo* bioassay, as predicted from its structure and physicochemical properties. This indicates that custom formulations can be used for macrocycles emerging from Discovery, where permeability may not be such an issue.

Randall Mrsny (University of Bath, United Kingdom) discussed how endogenous pathways in the GI epithelium can be exploited for efficient paracellular uptake of peptide and vesicular transcytosis of protein therapeutics. The introduction of the talk described the epithelial transport pathways of the intestinal barrier, which are selective and capacity-limited. A paracellular approach example recounted how the permeant inhibitor of phosphatase (PIP) peptides developed by Mrsny’s lab can open epithelial tight junctions by changing the myosin light-chain kinase and phosphatase enzyme activation balance. By understanding the enzymatic control of proteins in the tight junction, PIP peptide candidates have been produced by chemical synthesis, and they can deliver therapeutic peptides to the blood in preclinical animal models. Rational design using amino acid replacement and d-versions has led to lead candidates with good stability, permeating enhancing capacity, and low toxicity (Taverner et al., 2023). Increasing peptide potency as enhancers remains a challenge so that efficacy might eventually be achieved with lower concentrations of PIP peptide. The second approach pioneered by Mrsny’s lab involved mimicking a toxin derived from *Vibrio cholerae*, Cholix (Chx), which used transcytosis to enter the GI epithelia. The toxin was truncated to remove the known toxic elements, and then macromolecules were conjugated to the resulting protein to form a complex that could transcytose the epithelium, as nature intended. Human interleukin-10 is an anti-inflammatory protein with the potential for use in inflammatory GI conditions, so the challenge was to create a fused Chx–hI-10 conjugate (AMT-101, Fay et al., 2020) that could remain in the lamina propria for a local anti-inflammatory effect while avoiding systemic side effects. Mrsny described the results from a recent phase II trial of AMT-101 presented in an enteric tablet to treat inflammatory symptoms in the small intestine, where the safety profile upon repeat administration was acceptable along with the indication of some benefits in patients with pouchitis (NCT04741087). By including the IgG1 element, C_h_2, a new complex could also be created with cholix conjugated to human growth hormone (C_h_2–Chx–hGH), which altered the capacity to produce systemic blood levels by promoting exocytosis pathways across the basolateral membranes of GI epithelia. A resulting bioavailability of 5% was detected in rats for hGH (Taverner et al., 2025).

Douglas Johns (Merck Research Laboratories, United States) discussed the development of the oral macrocycle, MK-0616, for treating hypercholesterolaemia. This much-anticipated talk reviewed the discovery of the stable potent molecule, MK-0616, and its subsequent formulation in tablets containing the enhancer C10. A phase III trial was completed since the workshop, and a new drug application was submitted to the FDA in 2026. If approved, it will be the second oral therapeutic peptide product containing an enhancer other than SNAC, since C8 was included as a component of the Mycappsa® oily suspension in 2020. Johns reviewed how elevated levels of low-density lipoprotein cholesterol (LDL-C) in plasma can cause atherosclerosis and how lowering it can reduce the chances of coronary disease. This led to the nomination of PCSK9 as a Discovery target to inhibit. Three injectable PCSK9 inhibitors have been approved to date, which include two monoclonal antibodies and a small interfering ribonucleic acid (siRNA) conjugate, but Johns argued that these are expensive and painful to administer, and therefore, an oral PCSK9 inhibitor may help address the low uptake by patients. With knowledge of the structure of the LDL-C–PCSK9 receptor complex, the team surmised that small molecules might not achieve sufficient selectivity and potency at the target site, whereas a cyclic peptide could potentially achieve high target affinity and selectivity. The use of mRNA display selection provided screening of 10^14^ libraries of peptides and, moreover, unnatural amino acids were incorporated to confer improved properties on high-affinity macrocycle binders. From initial leads, structure-based design then drove optimisation of the structure–activity relationship, and this produced MK-0616 (enlicitide decanoate) (Tucker et al., 2021). This molecule had pM potency and was stable and soluble, but it was poorly permeable. Examination of the binding mode indicated mimicry of an approved antibody therapy. When orally-administered to cynomolgus monkeys in tablets formulated with a permeation enhancer, sufficient bioavailability was achieved to reduce plasma LDL-C, and this enabled clinical development. Johns then described the phase I, phase II, and topline phase III (CORALreef trial) data obtained for which Merck opted for C10 over Labrasol®-ALF as the enhancer. A key endpoint from the phase II trial was the reduction in plasma LDL-C from baseline to week 8 versus the placebo in patients with high cholesterol with daily tablets containing 20 mg–30 mg enlicitide with 180 mg C10 (Ballantyne et al., 2023). Phase III data from at-risk patients have just been published, which confirmed lower LDL-cholesterol than that achieved with placebo at 24 weeks for daily enlicitide tablets (Navar et al., 2026).

Sumit Arora, (J & J, United States) discussed some general principles regarding formulating oral peptides. Since their lead oral peptide product was under FDA review at the time of the meeting, he did not discuss the development of J & J’s oral macrocycle therapeutic to treat plaque psoriasis by blocking the IL-23 receptor. Icotrokinra (Icotyde®) (JNJ-77242113) was eventually approved as an oral formulation to treat psoriasis by the FDA in March 2026. The discovery path to the optimised potent and gut-stable high-affinity macrocyclic peptide binder (Fourie et al., 2024), and the subsequent phase III trials have been published (Gold et al., 2025). In describing how J & J approach the problems of oral peptide administration, Arora emphasised the importance of integrating molecule designs with formulation. He described numerous design methods using unnatural amino acids, cyclic and bicyclic structures, and backbone modifications to produce gut-stable and permeable peptides. A major challenge even for approved oral peptides is a negative food effect, which is poorly understood and may lead to a decline in patient adherence. Current efforts using the uFLux™ assay from pION can rank the interactions of lead peptides with bile, and this can direct point mutations in a peptide series to reduce such interactions and lead to peptide formulations that can be taken with food. Arora then discussed translational pathways for permeation enhancer–peptide combinations, noting the poor prediction from preclinical models to humans. Nonetheless, they continue to evaluate liquid-based formulations in rat models before moving to solid dose efforts in minipigs, dogs, and monkeys. Liquid enhancer formulations used in their rat gut instillation screens include C10, SNAC, sucrose laurate, Labrasol®-ALF, and the Peptelligence™ technology based on acyl carnitines. Selection is based on the performance in the rat model, compatibility with the peptide, enhancer potency, and capacity for regional GI release. An example was cited for a J and J peptide being formulated with C10, which was evaluated in dogs and went through to human trials, where the peptide tablet formulation with C10 produced seven times higher AUC than the control tablet under fasted conditions.

Takamitsu Ueto (Chugai Pharma, Japan) discussed Chugai’s programme to develop an oral macrocycle to access intracellular RAS targets in cancer and reviewed how the LUNA18 molecule reached phase I trials. Again, the potential of mid-size peptides to reach intracellular targets was cited as an advantage in contrast to monoclonal antibodies and small molecules. However, this is predicated on reasonable plasma membrane permeability being dialled into the peptide structure. Chugai also used mRNA display allied to *in silico* design to produce high-affinity RAS binders. The desirable features of lead candidates produced by the platform included having 9-11 residues, amide cyclization, cLog P of ∼12, and no polar amino acids. Optimisation produced LUNA18 (paluratide™, molecular weight 1438 Da), which is lipophilic and had good passive permeability across Caco-2 monolayers (Tanada et al., 2023; Matsuo et al., 2025). LUNA18 achieved intracellular access and showed anti-tumour activity in a mouse xenograft model. Remarkably, it achieved oral bioavailability values from the administration of solutions of over 21% in four animal species in the absence of permeation enhancers, including 26% in monkeys and 47% in dogs. Ueto then described the oral formulation programme for LUNA18, which presented solubilisation challenges. Since the aqueous solubility of LUNA18 crystals was low, they followed the cyclosporine (Neoral®) playbook by turning to lipid-based formulations, specifically self-emulsifying delivery systems (SEDDS). The lead molecule was especially soluble in lipophilic surfactants, where 20 mg/mL–30 mg/mL was achieved, which meant that they could conduct high-throughput screening using SEDDS libraries. The lead composition formulated with one lead peptide from the series comprised a SEDDS of oleic acid, propylene glycol (PG) monocaprylate, and Koliphor® EL. This formulation strategy was then optimised for mixtures with LUNA18, where the team discovered that this peptide also had good solubility in Labrasol ALF, triacetin, and polysorbate 80. SEDDS number 85 comprised oleic acid (15 mg/mL), PG monocaprylate (55 mg/mL), Kolliphor ®EL (29 mg/mL), and dl-α-tocopherol (1 mg/mL), which produced a solubility of 60 mg/mL. Examining dispersions in FaSSIF, SEDDS number 85 yielded an immediate uniform dispersion in 5 min, whereas another produced heterogenous micro-sized oily droplets with poor dispersion at 60 min. SEDDS number 85 also maintained solubility after *in vitro* digestion in FaSSIF with pancreatin. A PK study in rats with LUNA18 dispersed in SNEDDS formulation 85 in a size-9 capsule produced similarly high AUC values to an oleic acid solution from a 10 mg/kg peptide oral dose. SEDDS formulation 85 continued to impress in a PK study in monkeys compared to oleic acid-based solutions, and now this formulation is in phase I. This project illustrates that there are compromises in formulation strategies: in solving the permeability issue, a solubility problem then emerged, which had to be solved by lipid-based formulation.

Karsten Lindhardt (Biograil Ltd, Denmark) discussed alternatives for permeation enhancers for oral peptide administration, focussing on peptide-device combinations. Specifically, he updated on Biograil’s BIONND™ system, a device for the administration of peptides across the stomach. The principle of the process was to entrap a peptide in a size-00 capsule, which attached to the stomach wall, followed by the activation of a “flip over” spike to temporarily insert in the stomach wall. The intra-wall spike insertion is, therefore, part of a solid-dose formulation that can achieve blood levels on a par with SC administration. The BIONDD™ proof-of concept has been established in dogs, whereby 2.5 mg of a model peptide was entrapped in a fully dissolvable spike and achieved exposure and variability in plasma levels similar to those with SC administration (Skak et al., 2026 In press). Using endoscopy to track the performance of the tablet in the stomach, attachment was seen at ∼5 min and detachment at 24 h for all canine subjects assessed, along with accounting for all devices at 3 days of GIT transit. The system is also being evaluated with adalimumab, which is a greater challenge than peptides due to its much higher molecular weight. With a target dose of 150 mg per month, spikes could be entrapped with the required 5.1 mg for daily dosing, and these spikes achieved reliable insertions in isolated porcine stomach mucosae. In a study on dogs, the 2.5 mg and 5 mg oral doses of BIONDD™ system (N = 10 per group) produced PK profiles similar to those of SC administration of adalimumab (2.5 mg, N = 4), with relative bioavailability values of >70% for oral administration, with all devices actuating. Other candidates being assessed in the device include pegfilgrastrim and mRNA in lipid nanoparticles. Lindhardt concluded by saying that Biograil has initiated injection moulding manufacturing for scale-up and has a peptide candidate defined for GLP safety and a first-in-human study.

David Brayden (University College Dublin, Ireland) facilitated the roundtable discussion. The participants were Manuel Sanchez-Felix (Halozyme, United States), Stephen Buckley (Novo Nordisk, Denmark), Mo Masjedizadeh (Protagonist Company, United States), Neil Matthias (Bristol Myers Squibb), and Randy Mrsny (University of Bath, United Kingdom). They addressed the questions raised by the attendees, which are summarised as follows.

1. What are the factors to consider in starting an oral formulation programme for a new peptide? The panel answers included focussing on advantageous medicinal chemistry features; having a molecule with a reasonable molecular weight of below 5,000 Da, high potency, good stability, and a long half-life; and understanding the PK–PD relationship for what successful oral administration should look like
2. What are the best bioassay screens for permeability discrimination (PAMPA, Caco-2 monolayers, uFLUX™, organoids, Ussing chambers, pig tissue, or rat gut instillations)? Are any predictive, do they provide direction, or is it a process of trial and error? The panel answers understandably reflected individual biases and experiences. Each bioassay offers useful information in helping to answer specific questions, but each of them also has limitations. A general view was no one assay offers predictive capacity, but that when several are combined, there can be direction for what type of formulation can be animals. It was emphasised that much research now goes into excipient interactions with peptides in tablets and capsules and the release rates from the dosage forms. These are not evaluated by cell and tissue bioassays or rodent studies.
3. At what point do solid dosage forms and large animal studies come into preclinical formulation of peptides? The answers reflected a range of perspectives. Most of the Pharma panellists did not set too much store by rodent studies, which is perhaps an indication of many failures in translation to large animals. However, it was clear that programmes had to start somewhere, usually in rats, and that large animal studies cannot be used for extensive formulation screening due to costs and ethical issues. This is where new screening tools such as the robotic *ex vivo* porcine jejunal system presented by Thomas Von Erlach may offer a bridge to formulation from admixtures.
4. Can you offer a SWOT analysis on pigs, cynomolgus monkeys, and dogs? The panel favoured dogs over the other two models. Pigs have an exceptionally long gastroretentive time for solid dosage forms, and the stomach needs to be bypassed with duodenal administration. Macaques are expensive, are offered in small numbers by few facilities, and there are additional ethical considerations with them. Nonetheless, they were useful in the evaluation formulations in the Merck programme for MK-0616 before it went to phase I. Despite the GI tract differences to the human GI in terms of regional pH and transit times, dogs have proven to be a some of the good model in some programmes that progressed to human trials and appear to be the most widely used advanced preclinical large animal model for oral peptides.
5. Have we hit a ceiling on the performance of permeation enhancers, or could there be new ones that can outperform C10 and SNAC ? What considerations would justify pursuing new enhancers, and what are the risks? The panel was of the view that ‘no one size fits all’ and that new agents would need to offer substantial absorption benefits over SNAC and C10 to be further considered due to the regulatory risks and additional toxicology requirements. Nonetheless, new permeation enhancers are being discovered, and many are being repurposed from existing excipient solubilisers and from the food industry. Combinations of enhancers using different mechanisms of action are also being examined.
6. What data are required for nanotechnology to become competitive in this field? With a history of overpromise and many failures, the panel felt that nanotechnology needs to outperform standard permeation enhancers, have a simple scale-up and manufacture process, and a reliance on GRAS materials. There is also a gap in knowledge regarding the capacity to evade intestinal mucus and ensure epithelial uptake that can deliver the required plasma levels from a solid dosage form with highly loaded particles. Some panellists were of the view that nanotechnology could still have a role to play in targeting gut carriers and transporters because the science of particle synthesis, targeting ligands, and expression in the GI tract has improved considerably in the period since the earlier efforts.

## Data Availability

The original contributions presented in the study are included in cited references. Opinions and interpretations are expressed by the authors in personal capacities; further enquiries can be directed to the corresponding authors.
